# Neue Aspekte der Glukokortikoidsubstitution bei Nebennierenrindeninsuffizienz

**DOI:** 10.1007/s00108-021-01209-4

**Published:** 2021-12-03

**Authors:** Tina Kienitz, Gesine Meyer

**Affiliations:** 1 Endokrinologie in Charlottenburg, Stuttgarter Platz 1, 10627 Berlin, Deutschland; 2 Medizinische Klinik 1 – Schwerpunkt Endokrinologie, Diabetes und Stoffwechsel, Universitätsklinikum Frankfurt, Theodor-Stern-Kai 7, 60590 Frankfurt, Deutschland

**Keywords:** Hydrocortison, Adrenale Krise, Notfallausweis, COVID-19, Mineralokortikoide, Hydrocortisone, Adrenal crisis, Emergency card, COVID-19, Mineralocorticoids

## Abstract

**Hintergrund:**

Eine adäquate Anpassung der Glukokortikoidsubstitution an unterschiedliche Situationen ist essenziell für Leistungsfähigkeit und Lebensqualität von Patienten mit Nebennierenrindeninsuffizienz (NNRI). Sie dient darüber hinaus der Vermeidung lebensbedrohlicher adrenaler Krisen.

**Ziel der Arbeit:**

Verbesserung der Versorgung von Patienten mit Nebennierenrindeninsuffizienz.

**Material und Methoden:**

Selektive Literaturrecherche unter besonderer Berücksichtigung neuerer Studien.

**Ergebnisse:**

Eine optimale Glukokortikoidsubstitution hat das Ziel, die physiologischen Kortisolschwankungen möglichst genau nachzuahmen. Hier haben in den letzten Jahren Präparate mit veränderter Pharmakokinetik das Therapiespektrum erweitert. Im Vordergrund stehen eine adäquate Anpassung der Substitution in Stresssituationen sowie die Vermeidung und adäquate Behandlung adrenaler Krisen, die mit einer Inzidenz von 4,8 bis 8,3 Krisen pro 100 Patientenjahre auftreten und mit einer Mortalität von etwa 0,5 pro 100 Patientenjahre weiterhin eine tödliche Bedrohung darstellen.

**Schlussfolgerung:**

Zur Verhinderung lebensbedrohlicher Nebennierenkrisen ist eine Schulung von Patienten, Angehörigen und insbesondere auch medizinischem Personal notwendig.

**Zusatzmaterial online:**

Die Online-Version dieses Beitrags (10.1007/s00108-021-01209-4) enthält den vollständigen Notfallausweis für Patienten mit Nebennierenrindeninsuffizienz.

## Abstract (en)

**Background:**

Appropriate glucocorticoid dose adjustment in specific situations significantly impacts quality of life and performance of patients with adrenal insufficiency. It is also pivotal for the prevention of adrenal crisis.

**Objectives:**

Improving medical care for patients with adrenal insufficiency.

**Materials and methods:**

Selective literature research focussing on the most recent studies.

**Results:**

Optimal glucocorticoid substitution aims at closely mimicking physiological fluctuations of cortisol levels. In recent years glucocorticoid preparations with modified pharmacokinetics have expanded the therapeutic arsenal. Adrenal crises occur with an incidence of 4.8–9.3 crises per 100 patient years. With a mortality of 0.5 per 100 patient years adrenal crisis is a life-threatening event. Therefore, it is of the utmost importance to adjust glucocorticoid dose in situations with increased cortisol demand in order to prevent as well as appropriately treat adrenal crisis.

**Conclusions:**

To prevent life-threatening adrenal crisis, patients, their families and medical staff require training.

Grundlage der Therapie einer Nebennierenrindeninsuffizienz (NNRI) ist die Hormonersatztherapie mit Glukokortikoiden. Diese sollte die physiologischen Kortisolschwankungen im Tagesverlauf so gut wie möglich nachahmen. Erschwert wird die bedarfsgerechte Substitutionstherapie durch den erheblich gesteigerten Kortisolbedarf in Stresssituationen. Erfolgt die in solchen Situationen notwendige Anpassung nicht oder ist eine ausreichende Resorption oraler Glukokortikoide beispielsweise aufgrund eines gastrointestinalen Infekts nicht gewährleistet, besteht die Gefahr einer lebensbedrohlichen adrenalen Krise.

Die klinischen Zeichen der NNRI sind unspezifisch, was die Diagnosestellung erschwert. Von Bedeutung ist, die verschiedenen Formen der NNRI zu unterscheiden (Tab. 1). Die häufigste Form ist die iatrogene (tertiäre) NNRI. Bereits nach wenigen Tagen einer Therapie mit 20–30 mg Prednisolonäquivalent kann die Hypothalamus-Hypophysen-Nebennieren-Achse nachhaltig gestört werden. Insbesondere hochpotente Depotpräparate und eine unphysiologische abendliche Gabe bergen das Risiko einer längerfristigen Suppression des Regelkreises [8].

**Table 1:** 

Primäre NNRI	Sekundäre NNRI	Tertiäre NNRI
Irreversible Schädigung der Nebennierenrinde	ACTH-Mangel und konsekutiv Atrophie der Nebennierenrinde	Suppression der Hypothalamus-Hypophysen-Nebennieren-Achse
*Epidemiologie*		
Prävalenz: 93–144/1 Mio. [2, 5, 22]	Prävalenz: 150–280/1 Mio. [22, 26]	Prävalenz: ca. 0,5–2,0 % der Bevölkerung [8, 16]
Inzidenz: 5/1 Mio./Jahr [2, 5]	Inzidenz: 20/1 Mio./Jahr [26]	
*Mögliche Ursachen*		
Autoimmunadrenalitis	Tumoren der Hypophysenregion	Iatrogen (länger anhaltende Glukokortikoidtherapie)
Beidseitige Adrenalektomie	Operationen in der Hypophysenregion	Zustand nach endogenem Cushing-Syndrom
Adrenogenitales Syndrom	Bestrahlung der Hypophysenregion	
Infektionen (Tuberkulose, CMV, HIV, Mykosen)	Hypophyseninfarkt/Sheehan-Syndrom	
Beidseitige Nebenniereneinblutungen (Meningokokkensepsis)	Autoimmunhypophysitis	
Beidseitige Nebennierenmetastasen	Granulomatöse Erkrankungen (Sarkoidose, Histiozytose X)	
Adrenoleukodystrophie	Infektionen (tuberkulöse Meningitis)	
Genetische Ursachen	Schädel-Hirn-Trauma	
	Genetische Ursachen	

*ACTH* adrenokortikotropes Hormon, *CMV* Zytomegalievirus, *HIV* „human immunodeficiency virus“, *NNRI* Nebennierenrindeninsuffizienz

## Therapie

Grundlage der Therapie einer NNRI ist die Hormonersatztherapie mit Glukokortikoiden. Das am häufigsten eingesetzte Glukokortikoid in Deutschland ist Hydrocortison (HC). Übliche Tagessubstitutionsdosen in Deutschland liegen bei 10–30 mg [27]. Die Einnahme sollte in 2–3 Einzeldosen erfolgen mit etwa zwei Drittel der Dosis beim Aufwachen am frühen Morgen und einem Drittel 6–8 h später (beispielsweise HC 15-10-0-0 mg oder 10-10-5-0 mg). Patienten mit sekundärer NNRI benötigen in der Regel niedrigere Substitutionsdosen.

Eine Substitution mit Prednisolon ist möglich, jedoch müssen die längere Halbwertszeit und stärkere Glukokortikoidpotenz im Vergleich zu HC (etwa 1:5) beachtet werden. Üblich ist eine 1‑mal tägliche Einnahme von 3 bis 5 mg Prednisolon am Morgen [7]. Prednisolon hat gegenüber HC ein ähnliches kardiovaskuläres Risikoprofil [30], jedoch negative Einflüsse auf Low-density-Lipoprotein-Cholesterin-Werte [28] und Knochendichte [11].

Seit 2012 ist HC mit 2‑stufiger Wirkstofffreisetzung (Plenadren®, verfügbar als 5- und 20 mg-Tabletten) zur Therapie der NNRI bei Erwachsenen zugelassen. Die veränderte Pharmakokinetik beruht auf einer schnellen Freisetzung von HC aus der Hülle des Medikaments und einer verzögerten Freisetzung aus dem Kern. In der randomisierten, kontrollierten Zulassungsstudie konnte eine Verbesserung von Lebensqualität, Blutdruck und Glukosetoleranz erzielt werden. In weiteren, offenen klinischen Studien konnte die Verbesserung von Lebensqualität und glykometabolischem Profil bei Patienten mit primärer NNRI bestätigt werden [4, 24], während Daten zum metabolischen Profil von Patienten mit sekundärer NNRI divergieren [24, 32]. Die Tagestherapiekosten liegen deutlich über denen einer konventionellen Therapie mit HC.

Im Sommer 2021 wurde HC mit verzögerter Wirkstofffreisetzung (Efmody®, verfügbar als 5- und 10 mg-Tabletten) für Patienten ab 12 Jahren mit adrenogenitalem Syndrom zugelassen. Eine spezielle Formulierung von HC führt bei dem Medikament zu einer verzögerten Absorption. Die Einnahme erfolgt um 22 Uhr, die Wirkung entfaltet sich in den frühen Morgenstunden. Hierdurch lässt sich die Androgenbildung effektiver supprimieren. Zwar waren in der randomisierten Phase-III-Zulassungsstudie über 6 Monate keine Unterschiede hinsichtlich der Kontrolle der 17-Hydroxy-Progesteron(17-OHP)-Serumspiegel über 24 h zu sehen, die morgendlichen 17-OHP-Serumspiegel ließen sich jedoch mit dem neuen Präparat besser supprimieren. Auch war in der 1‑armigen Extensionsstudie eine Reduktion der HC-Tagesdosis möglich [21].

Übliche Tagessubstitutionsdosen liegen bei 10-30 mg Hydrocortison (HC)

Durch eine Pumpentherapie mit HC lässt sich die Glukokortikoidsubstitution dem physiologischen Profil am besten annähern. Mittels kontinuierlicher subkutaner HC-Infusion lässt sich eine verbesserte Lebensqualität erzielen [25]. Andere anthropometrische Parameter oder klinische Parameter zeigten jedoch gegenüber der 3‑mal täglichen oralen Einnahme keine Unterschiede [6]. Patienten mit nur geringer Einschränkung ihrer Lebensqualität scheinen nicht zu profitieren [12]. Insbesondere für Patienten mit primärer NNRI und stark fluktuierenden Energieniveaus während des Tages unter konventioneller Therapie könnte eine Pumpentherapie jedoch eine Option darstellen. In Deutschland ist der Einsatz nur im Rahmen klinischer Studien und individueller Einzelfallentscheidungen möglich.

## Die richtige Glukokortikoiddosis

Die endogene Kortisolsekretion folgt einer zirkadianen Rhythmik mit dem höchsten Spiegel in den frühen Morgenstunden (gegen 8 Uhr) und einem etwas kleineren Peak am frühen Nachmittag (gegen 14 Uhr). Am niedrigsten ist die Kortisolsekretion um Mitternacht. Stress, Infektionen oder Verletzungen erhöhen die endogene Kortisolproduktion um ein Vielfaches. Während einerseits eine chronische Übersubstitution vermieden werden muss, muss andererseits ein akut vermehrter Kortisolbedarf zwingend ausgeglichen werden. Die Güte der Glukokortikoidsubstitution lässt sich nicht anhand laborchemischer Parameter überwachen. Die Einstellung obliegt der klinischen Einschätzung sowie dem Suchen nach Zeichen und Symptomen der Über- bzw. Untersubstitution (Tab. 2).

**Table 2:** 

Übersubstitution	Untersubstitution
Gewichtszunahme	Gewichtsabnahme
Cushingoide Zeichen: Facies lunata, Hautatrophie, Muskelschwäche, nuchales und supraklavikuläres Fettpolster	Hypotonie und Schwindel
	Müdigkeit und Erschöpfung
Glukoseintoleranz/Diabetes mellitus	Übelkeit und Erbrechen
Osteopenie/Osteoporose	Hyponatriämie und/oder Hypoglykämie
Hypertonie	Hyperpigmentierung (bei primärer Nebennierenrindeninsuffizienz)

## Mineralokortikoide

Bei der primären NNRI muss zusätzlich eine Mineralokortikoidsubstitution mit 0,05–0,15 mg Fludrocortison 1‑mal täglich erfolgen. Anders als bei der Glukokortikoidsubstitution gibt es klinische Kontroll- und laborchemische Verlaufsparameter (Anstreben einer Normotonie, Serumnatrium und -kalium im Normbereich, Renin). Bei erhöhtem Blutdruck muss eine Reduktion, bei sehr hohen Temperaturen oder in der Schwangerschaft eine Steigerung der Fludrocortisondosis erwogen werden.

## Risiko einer adrenalen Krise

Eine akute Unterversorgung mit Kortisol ist ein potenziell lebensbedrohliches Krankheitsbild. Sie kann zu

- Hypotonie bis hin zum Schock,
- Elektrolytentgleisungen mit Hyponatriämie und Hyperkaliämie,
- Hypoglykämien,
- prärenalen Nierenfunktionseinschränkungen bis hin zum Nierenversagen sowie
- Bewusstseinseinschränkungen

führen.

Trotz der guten Behandlungsmöglichkeit sind Nebennierenkrisen weiterhin eine tödliche Bedrohung

Definiert wird eine adrenale Krise als akute Verschlechterung des Gesundheitszustands mit mindestens 2 der folgenden Symptome:

- Ausgeprägte Schwäche
- Übelkeit und/oder Erbrechen
- Hypotonie (systolischer Blutdruck < 100 mm Hg)
- Dokumentierte Hyponatriämie und/oder Hyperkaliämie und/oder Hypoglykämie

Es besteht die Notwendigkeit zur parenteralen Glukokortikoidgabe [1].

Die Inzidenz ist mit 4,8–8,3 Krisen pro 100 Patientenjahre höher als früher vermutet [15, 22, 29]. Das Risiko ist bei primärer NNRI mehr als doppelt so hoch wie bei sekundärer NNRI. Trotz der prinzipiell guten Behandlungsmöglichkeit stellen Krisen weiterhin eine tödliche Bedrohung dar mit einer Mortalität von etwa 0,5 pro 100 Patientenjahre [15]. Auslöser einer adrenalen Krise sind in Tab. 3 zusammengefasst.

**Table 3:** 

Auslöser	Häufigkeit (%)
Gastrointestinale Infekte	22–33
Fieberhafte Erkrankungen	17–24
Unzureichende Anpassung der Substitution bei einem operativen oder invasiven Eingriff	7–16
Intensive körperliche Belastung	7–8
Erhebliche psychische Belastung	4–6

## Vermeidung von Nebennierenkrisen

**Figure 1:**
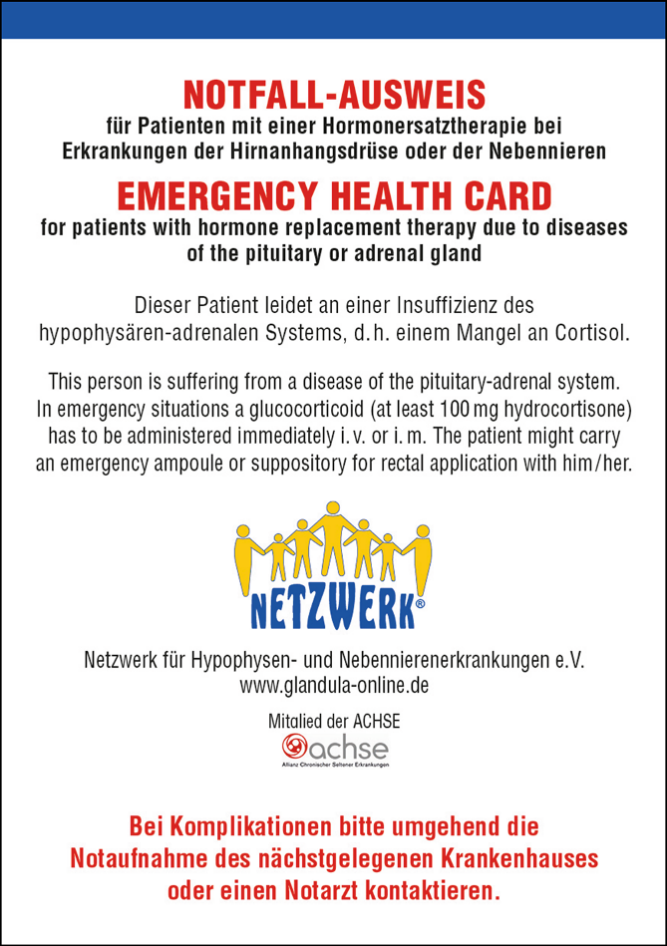

Um das Auftreten einer adrenalen Krise zu vermeiden, müssen Patienten ihre Substitutionsdosis eigenständig rasch und adäquat an den aktuellen Bedarf anpassen. Seit 2014 bietet die Deutsche Gesellschaft für Endokrinologie ein strukturiertes und einheitliches Curriculum zur Schulung von Patienten mit NNRI und ihrer Angehörigen an. Mittlerweile findet diese Schulung regelmäßig in vielen endokrinologischen Zentren statt und zeigt positive Auswirkungen [9, 10]. Alle Betroffenen müssen mit einem Notfallausweis (Abb. 1) ausgestattet werden, der die wichtigsten Informationen zum Vorgehen bei Erkrankung, Unfall und Operation sowie im Falle einer adrenalen Krise beinhaltet (Tab. 4).

**Table 4:** 

Situation	Empfehlung
Leichte Verletzungen	Gegebenenfalls zusätzliche Einnahme von 5 bis 10 mg HC
Anstrengende Aktivitäten über das Gewohnte hinaus	
Infekt mit leichtem bis mittlerem Krankheitsgefühl ohne Fieber oder deutliche Belastungssituation (starke körperliche Belastung, starker Schmerz, Zahneingriffe, kleinere ambulante Eingriffe, erhebliche psychische Belastung, z. B. bei Trauerfall, Prüfung, Hochzeit)	Tagesdosis verdoppeln, gegebenenfalls zusätzlich abends 5–10 mg HC
Akute Erkrankungen und/oder Fieber mit deutlichem Krankheitsgefühl	Tagesdosis verdreifachen oder 30-20-10 mg HC (bei Tagesdosis ≤ 20 mg)
	Dringend ärztliche Hilfe holen
Anhaltendes Erbrechen und/oder Durchfall oder hohes Fieber (> 39 °C) mit schwerem Krankheitsgefühl	100 mg HC (oder anderes Glukokortikoid) parenteral
	*Sofort* ärztliche Hilfe holen!
Operationen (stationär, Vollnarkose)	*Operationstag*: 100 mg HC i.v. als Bolus vor Narkoseeinleitung, gefolgt von 100 bis 200 mg/24 h i.v.
	*Postoperativ*: 100 mg/24 h i.v., bis Patient essen und trinken darf, dann Umstellung auf doppelte bis 3‑fache Tagesdosis für 24–48 h, weitere Reduktion nach Verlauf und Klinik

*HC* Hydrocortison

Bei invasiven Eingriffen sowie während einer Entbindung ist eine entsprechend hoch dosierte parenterale Substitution durch den Behandler zu gewährleisten [1, 7]. Für kleinere ambulante Eingriffe in Lokalanästhesie, wie zahnärztliche Eingriffe oder Exzisionen von Hautveränderungen, genügt in aller Regel eine Verdopplung der oralen Dosis (Tab. 4).

Auch die für eine Koloskopie notwendigen Abführmaßnahmen können prinzipiell zur Entstehung einer adrenalen Krise führen. Es sollten daher isoosmotische Abführpräparate mit einem möglichst geringen Risiko von Flüssigkeits- und Elektrolytverschiebungen zum Einsatz kommen. Insbesondere bei älteren und multipel vorerkrankten Patienten sind eine Durchführung der abführenden Maßnahmen unter stationärer Überwachung und eine parenterale Gabe von HC (beispielsweise 50 mg i.v. alle 8 h ab Beginn der Abführmaßnahmen) zu empfehlen.

## Behandlung der adrenalen Krise

Der Übergang von einer drohenden zu einer manifesten adrenalen Krise verläuft fließend. Bereits eine drohende Krise ist ein medizinischer Notfall, der rasches Handeln erfordert. Die wichtigste therapeutische Maßnahme ist die intravenöse Zufuhr von Glukokortikoiden und Flüssigkeit unter engmaschigem Monitoring.

Rasches Handeln rettet bei einer drohenden oder bereits manifesten adrenalen Krise Leben und gefährdet die Patienten auch dann nicht, wenn die Situation anfangs überschätzt wurde und keine adrenale Krise vorliegt.

Akuttherapie (nach [7]):

- 100 mg HC i.v. als Bolus
- Gefolgt von 200 mg/Tag als 24 h-Dauerinfusion oder regelmäßige Gabe eines Bolus von 50 bis 100 mg alle 6 h
- Wenn HC nicht verfügbar ist: 25 mg Prednisolon i.v. als Bolus, gefolgt von regelmäßigen Boli. Cave: Bei Verwendung von Glukokortikoiden ohne ausreichende Mineralokortikoidwirkung bei primärer NNRI zusätzliche Applikation von 0,05 bis 0,15 mg Fludrocortison p.o. pro Tag
- Infusion von balancierter Elektrolytlösung oder 5 %iger Glukoselösung in isotonischer Kochsalzlösung mit einer anfänglichen Infusionsrate von 1 l/h, Anpassung der Infusionsrate nach Stabilisierung
- Intensivmedizinische Überwachung mit engmaschigem hämodynamischem Monitoring
- Engmaschige Elektrolytkontrollen (insbesondere Natrium, Kalium, Glukose) und gegebenenfalls Ausgleich

Das Erleben einer adrenalen Krise und auch bereits die Angst vor dieser Komplikation belasten die Betroffenen erheblich [23]. Verstärkt wird dies durch eine verzögerte Erkennung [17] und Therapie [13] durch medizinisches Fachpersonal. Leider berichten Patienten nicht selten von Situationen, in denen auch nach Vorlage des Notfallausweises eine adäquate Hilfe erst nach deutlicher Zeitverzögerung, teilweise auch gar nicht erfolgte.

## Nebennierenrindeninsuffizienz und COVID-19

Die Bedrohung durch eine potenziell lebensbedrohlich verlaufende Infektion mit dem „severe acute respiratory syndrome coronavirus 2“ (SARS-CoV-2) führt seit Beginn der Pandemie bei Patienten mit NNRI zu einer teilweise erheblichen zusätzlichen Belastung [20]. Es ist wenig darüber bekannt, ob das Risiko einer Ansteckung und/oder des schweren Verlaufs einer „coronavirus disease 2019“ (COVID-19) bei vorliegender NNRI signifikant erhöht ist. Bei Patienten mit primärer NNRI konnte eine verminderte zytotoxische Aktivität natürlicher Killerzellen gezeigt werden, die insbesondere für die Erkennung und Eliminierung virusinfizierter Zellen benötigt werden. Als Ursache dieser Beeinträchtigung des angeborenen Immunsystems wird am ehesten die trotz aller Bemühungen letztlich unphysiologisch bleibende Substitution mit Glukokortikoiden vermutet, die zu einer veränderten peripheren *CLOCK*-Genregulation in den Immunzellen führt [3]. Vor diesem Hintergrund erscheinen ein höheres Risiko viraler Infektionen und auch ein schwererer Verlauf solcher Infektionen bei NNRI durchaus möglich – auch bei Gabe einer physiologischen Substitutionsdosis. COVID-19 kann darüber hinaus eine adrenale Krise auslösen, insbesondere bei deutlichen Symptomen, hohem Fieber und/oder gastrointestinalen Symptomen. Wie bei anderen Infektionen ist daher eine rasche Dosisanpassung und gegebenenfalls parenterale Substitution auch bei COVID-19 von höchster Wichtigkeit.

Patienten mit Nebennierenrindeninsuffizienz sollten gegen COVID-19 geimpft werden

Patienten mit Nebennierenrindeninsuffizienz sollten gegen COVID-19 geimpft werden [19]. Eine Beeinträchtigung der Impfwirkung ist unter den üblichen Substitutionsdosen nicht zu erwarten. Valide Daten hierzu liegen bislang nicht vor, bei gängigen Impfungen werden jedoch bis zu einer Tagesdosis von 10 bis 20 mg Prednisolon keine relevanten Beeinträchtigungen des Impferfolgs beobachtet. Die Impfung sollte unter der üblichen Substitutionsdosis erfolgen. Im Falle des Auftretens einer Impfreaktion, insbesondere bei Fieber, muss die Dosis analog zu den Empfehlungen für Infekte passager erhöht werden [18].

## Fazit für die Praxis

- Bei unspezifischen Symptomen wie Müdigkeit, Erschöpfung, Gewichtsverlust, Hypotonie oder Hyponatriämie gilt es, eine Nebennierenrindeninsuffizienz (NNRI) differenzialdiagnostisch auszuschließen.
- Am häufigsten ist die iatrogene NNRI nach Pharmakotherapie mit Glukokortikoiden.
- Eine optimale Glukokortikoidsubstitution hat das Ziel, die physiologischen Kortisolschwankungen möglichst genau nachzuahmen. In diesem Zusammenhang haben in den letzten Jahren Präparate mit veränderter Pharmakokinetik das Therapiespektrum erweitert.
- Nicht wenige Patienten berichten von Situationen, in denen auch nach Vorlage des Notfallausweises eine adäquate Hilfe erst nach deutlicher Zeitverzögerung, teilweise auch gar nicht erfolgte.
- Zur Verhinderung lebensbedrohlicher Nebennierenkrisen ist eine Schulung von Patienten, Angehörigen und auch medizinischem Personal notwendig.
- Patienten mit NNRI sollten alle empfohlenen Impfungen erhalten – auch gegen COVID-19.

## Supplementary Information




