# Supplementary material for: New aspects of glucocorticoid substitution in adrenal insufficiency
Source: Internist (Berl). 2021 Dec 3;63(1):12–7. [Article in German] doi: 10.1007/s00108-021-01209-4 (PMC8640964; doi:10.1007/s00108-021-01209-4)
Supplement: Supplementary file 1 [file 108_2021_1209_MOESM1_ESM.pdf]

Wichtige Informationen

- Impfungen sind grundsätzlich entsprechend den Empfehlungen der STIKO uneingeschränkt möglich, bei fieberhafter Impfreaktion sollte die Glucocorticoiddosis gesteigert werden.
- Hydrocortison wird u. a. in der Leber durch das Enzym CYP3A4 abgebaut. Bei längerfristiger Einnahme von Medikamenten, die die Aktivität von CYP3A4 erhöhen (z. B. Carbamazepin, Phenytoin, Johanniskrautextrakt, Mitotane), muss ggf. die Hydrocortison-Dosis gesteigert werden. Bei Medikamenten, die den Abbau verlangsamen (z. B. Fluconazol, Voriconazol, Clarithromycin, Aprepitant, Verapamil, Cimetidin, HIV-Proteaseinhibitoren), muss ggf. die Dosis reduziert werden. Die Dosisanpassung sollte jeweils mit dem behandelnden Endokrinologen besprochen werden.
- Im Rahmen einer Schwangerschaft muss die Corticoid-Dosis individuell angepasst werden und eine engmaschige endokrinologische Betreuung der Patientin/des Patienten gewährleistet sein.

Zeichen eines drohenden Corticoid-Mangelzustandes

- Übelkeit, Erbrechen, Bauchschmerzen
- Unterzuckerungen (Kaltschweißigkeit, Herzrasen, Hunger)
- niedriger Blutdruck, Schwindel
- Antriebsarmut, Reizbarkeit oder Apathie
- Gewichtsabnahme
- Flüssigkeitsmangel
- langsamer Herzschlag
- Kreislaufkollaps
- Schock mit tiefer Bewusstlosigkeit
- Verschiebung der Blutsalze

Die Behandlung des Patienten erfolgt durch

Hausarzt

betreuende endokrinologische Institution

Datum/Unterschrift behandelnder Arzt

Für den Zoll

Diese Person führt zur Aufrechterhaltung einer Hormonersatztherapie ein Spritzenbesteck und/oder einen Pen als Injektionshilfe sowie Hydrocortison und/oder andere Medikamente mit sich.

For customs

This person is undergoing continuous hormone replacement therapy, and for this reason is carrying an injection device/pen, hydrocortisone and/or other drugs.

Netzwerk für Hypophysen- und Nebennierenerkrankungen e.V.

Die Erstellung des Ausweises erfolgte in Zusammenarbeit mit der Deutschen Gesellschaft für Endokrinologie

Fachliche Beratung durch die Sektion Nebenniere der Deutschen Gesellschaft für Endokrinologie

und

mit der Deutschen Gesellschaft für Kinderendokrinologie und -diabetologie

Aktualisiert im Juni 2020.

NOTFALL-AUSWEIS

für Patienten mit einer Hormonersatztherapie bei Erkrankungen der Hirnanhangsdrüse oder der Nebennieren

EMERGENCY HEALTH CARD

for patients with hormone replacement therapy due to diseases of the pituitary or adrenal gland

Dieser Patient leidet an einer Insuffizienz des hypophysären-adrenalen Systems, d. h. einem Mangel an Cortisol.

This person is suffering from a disease of the pituitary-adrenal system. In emergency situations a glucocorticoid (at least 100 mg hydrocortisone) has to be administered immediately i. v. or i. m. The patient might carry an emergency ampoule or suppository for rectal application with him/her.

Netzwerk für Hypophysen- und Nebennierenerkrankungen e.V.
www.glandula-online.de

Mitglied der ACHSE

Bei Komplikationen bitte umgehend die Notaufnahme des nächstgelegenen Krankenhauses oder einen Notarzt kontaktieren.

Foto

Name / surname Vorname / first name

Geburtsdatum / date of birth

Anschrift / address

Telefon / phone

im Notfall zu benachrichtigen / in case of emergency to be informed

Bitte führen Sie diesen Ausweis stets bei sich!

Diagnose

Dauerhafte Substitution (Dosis/Tag)

1.
Glucocorticoid

2.
Mineralocorticoid (nur für Patienten mit primärer NN-Insuffizienz)

3.
L-Thyroxin

4.
Sexualhormon

5.
Somatotropin

6.
Desmopressin

weitere wichtige Medikamente

**Situationen, in denen ein Corticoid-Mangel droht, der mit der Gabe von Hydrocortison (oder im Notfall mit jedem anderen Glukokortikoid) substituiert werden muss:**	
leichte Verletzungen, anstrengende Abendveranstaltungen, Aktivität über das Gewohnte hinaus	**ggf. zusätzliche Einnahme von 5–10 mg Hydrocortison**
Infekt mit leichtem bis mittlerem Krankheitsgefühl ohne Fieber oder deutliche Belastungssituation (starke körperliche Belastung), starker Schmerz, erheblicher Stress (Trauerfall, Prüfung, Hochzeit), Zahneingriffe, kleine ambulante Eingriffe	**Tagesdosis verdoppeln, ggf. zusätzlich abends 5–10 mg Hydrocortison**
akute Erkrankung und/oder Fieber mit deutlichem Krankheitsgefühl	**Tagesdosis verdreifachen oder 30-20-10 mg Hydrocortison (bei Tagesdosis ≤20 mg Hydrocortison/d)** **Dringend ärztliche Hilfe einholen!**
anhaltendes Erbrechen/Durchfall oder hohes Fieber (>39 °C) mit schwerem Krankheitsgefühl	**100 mg Hydrocortison (oder anderes Glucocorticoid) als Selbstinjektion in den Muskel, unter die Haut oder als Infusion** **SOFORT ärztliche Hilfe einholen!**
Operation (stationär, Vollnarkose)	OP-Tag: 100 mg i. v. als Bolus vor Narkoseeinleitung, gefolgt von 100–200 mg/24 h i. v. Nach OP: 100 mg/24 h i. v., bis Patient essen/trinken darf, dann umstellen: doppelte orale Tagesdosis für 24–48 h, dann Reduktion je nach Klinik

Hydrocortison wirkt nur 6–8 Stunden und muss deshalb bei länger anhaltenden Problemen mehrfach täglich gegeben werden! Im Zweifelsfall IMMER großzügig Hydrocortison einnehmen! Erst handeln, dann denken! Nach jeder Selbstinjektion Arzt/Notarzt informieren oder Krankenhaus aufsuchen, NOTFALLAUSWEIS vorlegen und idealerweise den letzten Arztbrief mitnehmen.

**Anpassung für Kinder und Jugendliche**	
**leichtgradige** psychische oder körperliche Beanspruchung z. B.: Wettkampf, Turnier, lange Wandertour, lange Klausur wie Abi-Prüfung oder Abschlussprüfung, leichte Zahnarztbehandlung (z. B. Füllung), emotionaler Stress (Trauerfall, Hochzeit)	Zusätzliche Einnahme einer Einzeldosis, etwa der Mittags- oder Abenddosis entsprechend vor dem Ereignis
**leichter Infekt** mit Temperatur <38,5 °C und geringem Krankheitsgefühl	Tagesdosis verdoppeln
Erkrankungen mit Fieber >38,5 °C	>38,5 °C Dosis verdreifachen, >39,5 °C Dosis vervierfachen, Dosiserhöhung bis zur Genesung, danach innerhalb von 1–2 Tagen Rückkehr auf die Standarddosis.
**bei schwerem Krankheitsgefühl** (unabhängig von der Temperatur), Reduktion des Allgemeinzustandes oder Änderung des Bewusstseins, Schock, Trauma	i. m. oder s. c. Gabe von Hydrocortison: Säuglinge: 25 mg; Kindergarten- und Grundschulalter: 50 mg, Jugendliche: 100 mg (alternativ: Zäpfchen, z. B. Rectodelt 100 mg, **nicht bei Durchfall!**). Nach der Injektion **ärztlich vorstellen!** Ggf. Fortsetzung der Hydrocortisontherapie 100 mg/m²/24h i. v. und Infusionstherapie (NaCl 0,9 %, Glukose)
falls orale Einnahme nicht möglich (z. B. bei **Magen-Darminfektion mit Erbrechen**)	i. m. oder s. c. Gabe von Hydrocortison: Säuglinge: 25 mg; Kindergarten- und Grundschulalter: 50 mg, Jugendliche: 100 mg (alternativ: Glukokortikoid-Zäpfchen, z. B. Rectodelt, 100 mg **nicht bei Durchfall!**). Nach der Injektion **ärztlich vorstellen!**
**Perioperative Hydrocortisontherapie** grundsätzlich sollten operative Eingriffe möglichst stationär und nicht ambulant erfolgen und die Rücksprache mit einem pädiatrischen Endokrinologen erfolgen	siehe AW/MF-Leitlinie Nebenniereninsuffizienz
